# Jugular Vein Thrombosis after Dental Extraction, from Lemierre’s Syndrome to Behcet’s Disease

**Published:** 2018-10

**Authors:** Alireza Bagheri, Maryam Mansour

**Affiliations:** Department of Internal Medicine, Hamadan University of Medical Sciences, Hamadan, Iran

**Keywords:** Behçet disease, Lemierre’s disease, Jugular vein thrombosis, Dental extraction

## Abstract

In this report, we describe a 48-year old non-smoker man who presented with dyspnea, light headedness, plethoric facies, neck swelling and swollen collateral veins on the front of the chest wall after extracting his molar tooth due to dental caries and tooth pain. Right internal jugular vein thrombosis was seen on the neck CT angiography. Lemierre’s disease was suspected and systemic antibiotics in addition to anticoagulant were started. Two months later the patient presented with characteristic genital and oral aphthous ulcers. A final diagnosis of Behçet vasculitis was made and the patient received high dose of immunosuppressive therapy. Dental extraction in Behçet disease may cause the disease flare up and large vessel thrombosis.

## INTRODUCTION

Lemierre’s syndrome is a septic thrombophlebitis of the internal jugular vein after an oropharyngeal infection, mostly caused by Fusobacterium, a normal human microflora of the oropharynx. Treatment of Lemierre’s syndrome is a long term of appropriate antibiotics therapy (1). Behcet’s Disease (BD), is a multisystem vasculitis with unknown etiology, diagnosed clinically and characterized by triad of recurrent oral aphthous ulcers, genital sores and relapsing uveitis, in young inhabitant along the ancient Silk Road (2). Vascular involvement is one of the major causes of morbidity and mortality in BD (3). Treatment of BD vasculitis involves immunosuppression and anticoagulant therapy (4). Meticulous history taking and physical examination is necessary to establish the correct diagnosis and initiate appropriate therapy.

## CASE SUMMARIES

A 48- year old non-smoker man, a known case of type II diabetes mellitus, was referred to our hospital with a two week history of dyspnea on exertion, cough and lightheadedness. His symptoms started after extracting his molar tooth due to dental caries and tooth pain. His dyspnea was aggravated on bending forward. He did not report dysphagia, hoarseness or drooling and gave no history of other illnesses. On physical examination he had soft tissue neck swelling, plethoric facies, prominent jugular vein, swollen collateral veins on the front of the chest wall, and acanthosis nigricans. He did not have fever, lymphadenopathy, dysphonia or focal neurological deficits. In oral cavity, there was diffuse gingivitis and the scar of the recent upper molar extraction. Auscultation of his lungs was clear and his heart sounds were without murmur. He had no edema in his extremities or any joint findings.

Upon arrival, he had a white blood cell count of 7600 with 88% neutrophils and 12% lymphocyte and erythrocyte sedimentation rate of 50 mm/hr. His blood culture was negative.

**Figure 1.**
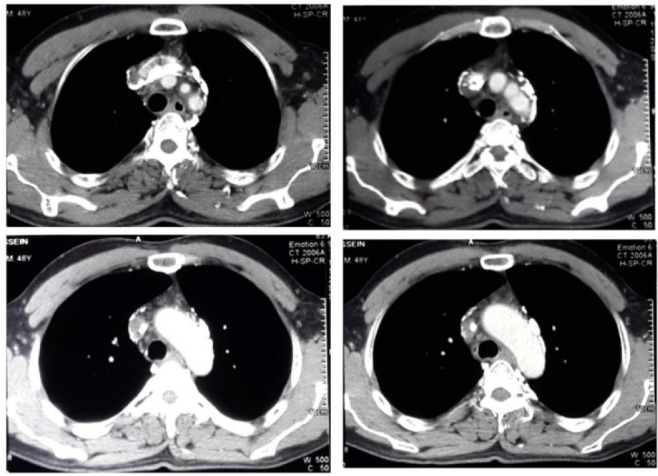
Chest CT scan with IV contrast shows irregular filling defect and thrombosis in the subclavian vein and superior vena cava

**Figure 2.**
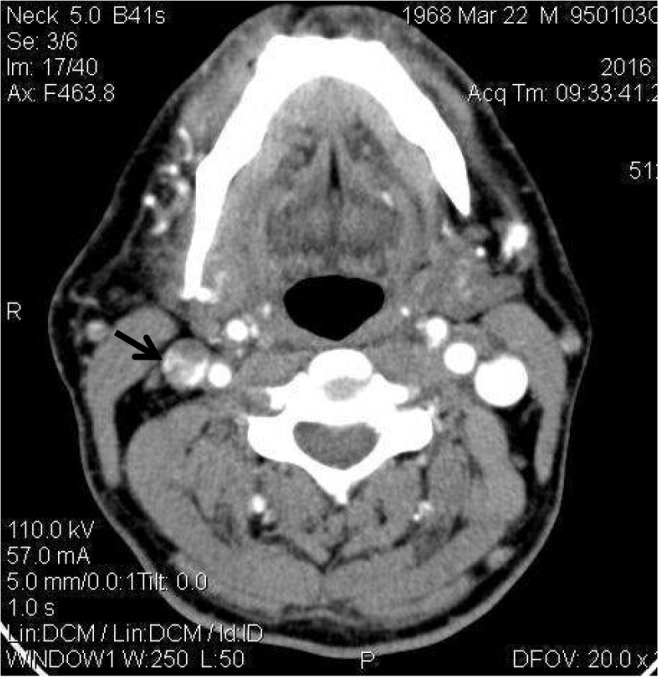
Axial veiw of the neck CT scan with IV contrast shows thrombosis in the right jugular vein (arrow)

Chest CT-scan showed thromboses of brachiocephalic vein and superior vena cave (Figure 1). In CT angiography of his neck, thrombosis of right internal jugular vein was reported (Figure 2) and anticoagulant therapy was started. A brain magnetic resonance venography did not show any sinus thrombosis. Thrombophilia screening result was negative.

Due to tooth pain and dental surgery just before thrombosis, Lemierre’s disease was suspected and systemic antibiotics were started. Maxillofacial surgeon and ENT consultations did not reveal any infection site. He was discharged with oral antibiotic and warfarin after three weeks.

**Figure 3.**
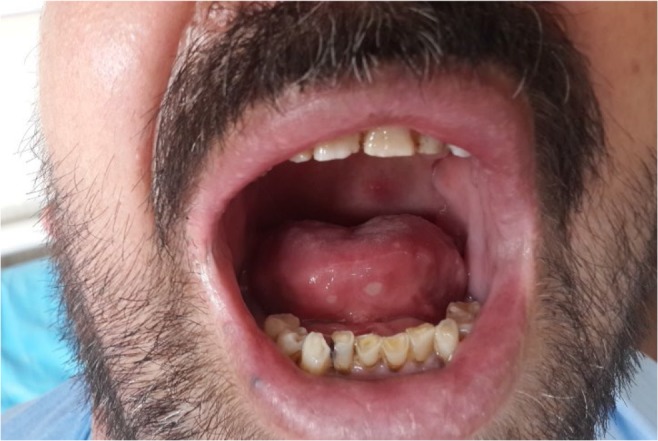
Two aphthous ulcer in the tip of the tongue

Upon further examination of the patient in follow up, the patient neck swelling, plethoric facies and prominent upper chest collateral veins did not improve and the characteristic oral aphthous (Figures 3) and genital aphthous ulcers appeared. On the revised history taking, he admitted recurrent and longstanding history of prior aphthous ulcers for 5 years. There was also folliculitis on the chest and cutaneous nodules in the forearms. Ophthalmologic examination did not show eye involvement. Based on the International Criteria for BD bipolar aphthous ulcers, skin lesions and central vein thrombosis, the diagnosis of BD was made and high dose of methyl prednisolone was started with monthly cyclophosphamide pulses for six months.

The patient was followed up for 30 months. Meanwhile he continued anticoagulant therapy with warfarin. He did not have any flare up of BD. Although physical signs of Superior Vena Cava (SVC) syndrome were still evident in the patient.

## DISCUSSION

Behcet’s Disease (BD), a multisystem vasculitis with unknown etiology, was first characterized by triad of recurrent oral aphthous ulcers, genital sores and relapsing uveitis, in young inhabitant (2). Cutaneous lesions (pseudofolliculitis, erythema nodosum, and skin aphthosis), joint (arthralgia, arthritis, and ankylosing spondylitis), vascular (arterial thrombosis, large vein thrombosis, and phlebitis), neurological (meningoencephalitis and seizures), gastrointestinal (intestinal mucosa aphthous ulcers, chronic diarrhea, and proctorrhagia) and pulmonary (vasculitis, embolism, fibrosis, and pleurisy) involvements have been frequently described in BD. Clinical presentation and incidence rate of various organ involvement differs slightly depending on the ethnicity and the geographic area (5).

Although BD is more prevalent in the ancient Silk Road countries, it has been reported in other countries due to immigration. Diagnosis of BD is based on the clinical manifestation. In the International Criteria for BD, ocular lesions, genital and oral aphthosis get 2 points for each and skin lesions, neurologic manifestations, vascular manifestations and positive pathergy test (if done) get one point for each. Scoring ≥ 4 indicates BD (5).

Vascular involvement is one of the major causes of morbidity and mortality in BD (3).

In a large cohort study of 6500 patients with BD in Iran over a period of 35 years, vascular involvement was detected in 539 cases (8.3%). Phlebitis, mainly in lower limbs, were seen in 5.7%, superficial phlebitis in 2.2%, large vein thrombosis (vena cava) in 1.1%, arterial thrombosis in 10 cases (0.154%), aneurysm in 31 cases (0.5%), and pulse weakness in three cases (0.046%) (6). In a study from Turkey, out of 2147 patients with BD, 361 (16.8%) had vascular involvement, 29 patients had occlusion of superior vena cava, 1 patient thrombosis of jugular vein and 1 had cerebral sinus thrombosis (7). Although vascular involvement in BD is more prevalent in youth and male gender (3, 6), other risk factors have not been determined. Disease modifying drugs such as pulse methylprednisolone, cyclophosphamide and azathioprine is usually added to anticoagulant in the treatment of large vessel thrombosis in BD (4).

Recurrent painful oral ulceration may limit regular oral hygiene habits (8). In addition, poor oral health, poor prognosis for the natural dentition, increased number of extracted teeth due to multiple carious lesions, changes in oral pH, colonization with oral micro-organisms such as streptococcus, and changing the properties of saliva have been reported more in BD than healthy subjects (9, 10). Although poor oral health and hygiene has been linked with the course of BD, and efforts to maintain a healthy oral hygiene may be considered as a part of BD therapy (8), dental and periodontal therapies could be associated with a flare-up of oral ulcers in the short term (11).

In review of the literature, we found two cases of BD flare up with large vessel thrombosis after tooth extraction. Mizushima et al. reported Iliac vein thrombosis one day after dental extraction in a young female BD patient (12). Hamza reported leg thrombosis, one day after dental extraction in a young male BD patient (13). There had been also reports of two cases of neuro-BD flare up in few days after molar tooth extraction (14, 15).

Wagner et al. also reported neck soft-tissue swelling and fever after tonsillectomy in a known case with prior diagnosis of BD, which was proven to be leukocytoclastic small vessel vasculitis and perivasculitis without infection source, compatible with a relapse of BD. The neck swelling responded well with immunosuppressive therapy (16).

In conclusion, large vessel thrombosis is a rare manifestation of the BD with significant morbidity. A careful history about recurrent aphthous lesion and other criteria should be considered in any patient with unusual thrombosis to initiate appropriate treatment. Dental surgery (as in our patient) has been reported as a trigger of large vein thrombosis and Behcet’s vasculitis flare up. Our case showed SVC syndrome due to large vessel thrombosis subsequent to BD flare after a dental procedure. Increasing the corticosteroid dosage might be advised before oro-dental surgery in any stable BD.
